# Eyeblink Classical Conditioning and Post-Traumatic Stress Disorder – A Model Systems Approach

**DOI:** 10.3389/fpsyt.2015.00050

**Published:** 2015-04-08

**Authors:** Bernard G. Schreurs, Lauren B. Burhans

**Affiliations:** ^1^Blanchette Rockefeller Neurosciences Institute, West Virginia University, Morgantown, WV, USA; ^2^Department of Physiology and Pharmacology, West Virginia University, Morgantown, WV, USA

**Keywords:** conditioning-specific reflex modification, explicitly unpaired, extinction, reflex modification, rabbit nictitating membrane response, virtual reality

## Abstract

Not everyone exposed to trauma suffers flashbacks, bad dreams, numbing, fear, anxiety, sleeplessness, hyper-vigilance, hyperarousal, or an inability to cope, but those who do may suffer from post-traumatic stress disorder (PTSD). PTSD is a major physical and mental health problem for military personnel and civilians exposed to trauma. There is still debate about the incidence and prevalence of PTSD especially among the military, but for those who are diagnosed, behavioral therapy and drug treatment strategies have proven to be less than effective. A number of these treatment strategies are based on rodent fear conditioning research and are capable of treating only some of the symptoms because the extinction of fear does not deal with the various forms of hyper-vigilance and hyperarousal experienced by people with PTSD. To help address this problem, we have developed a preclinical eyeblink classical conditioning model of PTSD in which conditioning and hyperarousal can both be extinguished. We review this model and discuss findings showing that unpaired stimulus presentations can be effective in reducing levels of conditioning and hyperarousal even when unconditioned stimulus intensity is reduced to the point where it is barely capable of eliciting a response. These procedures have direct implications for the treatment of PTSD and could be implemented in a virtual reality environment.

## Introduction

People exposed to trauma who suffer flashbacks, bad dreams, numbing, fear, anxiety, sleeplessness, hyper-vigilance, hyperarousal, or an inability to cope comprise the 15–25% who suffer from post-traumatic stress disorder (PTSD) (1–3). There is a crucial need to know how responding to stressful events changes as a function of trauma for patients who suffer from PTSD and particularly combat-related PTSD – a condition that can be resistant to behavioral and drug therapy (2, 4, 5). PTSD is the most common psychiatric condition for which veterans seek services (6, 7). PTSD among veterans may be 3 times higher than in the general population, although it may be 30 times higher in combat veterans (8). Even these numbers may be underestimates due to under-reporting of mental disorders in active duty personnel because of perceived weakness, loss of confidence, stigma, and threat to career posed by a need for mental health services (6, 9–11). Adding further concern are recent findings that PTSD can lead to an increased risk of dementia (12, 13) and PTSD symptoms can last more than 15 years (14). Despite some progress in diagnosing and treating PTSD in civilians, treating veterans is less successful (5, 15, 16), and PTSD among veterans results in increased death (17, 18) including suicide (18, 19). It is clear every effort, including better animal modeling, needs to be made to improve our understanding and treatment of PTSD.

Researchers have developed a range of animal models of PTSD (3, 20–29). Although animal models cannot capture all the aspects of a human disorder, they are invaluable for developing and testing potential treatments, especially when a model expresses more than one phenotype of PTSD (30–33). However, many of the current animal models of PTSD have limitations. First, they focus on the fear associated with trauma (fear conditioning) without assessing or treating the hyperarousal caused by trauma or they focus on stress-induced hyperarousal without assessing or treating fear conditioning. Second, the majority of animal models rely on group data, and it is clear that not everyone exposed to trauma develops PTSD (2, 13, 30, 34, 35). In fact, depending on the population and on the type of trauma, only 5–25% of exposed people develop PTSD (1–3).

We have developed an animal model of PTSD in which conditioning and hyperarousal can both be extinguished (36). The model is based on observations that the eyeblink response becomes exaggerated as a function of classical conditioning (37–43). The exaggerated response occurs when the eliciting stimulus such as an air puff or periorbital electrical stimulation is tested by itself, and this form of hyperarousal is termed conditioning-specific reflex modification (CRM). CRM is detected by comparing responses to a range of unconditioned stimulus (US) intensities by themselves before and after classical conditioning. This phenomenon has been observed by others in rabbit eyeblink conditioning (44, 45) and in rat eyeblink conditioning (46). We now have strong evidence we can “treat” CRM as well as extinguish conditioned responses (CRs) to stimuli associated with the US. Importantly, high levels of CRM only occur in 15–25% of rabbits exposed to eyeblink classical conditioning (EBCC) – levels that are consistent with the incidence of PTSD (2, 3, 35).

## Eyeblink Classical Conditioning

### EBCC in humans

The history of human EBCC dates back to German studies beginning in 1899 and described by Woodruff-Pak and Steinmetz (47) who referenced an exhaustive bibliography of over 500 human EBCC studies from 1899 to 1985 compiled by Gormezano (48). EBCC in the United States was pioneered by Cason in 1922 using electric shock as the US (49). EBCC was then expanded upon by Hilgard in a subsequent series of studies in the 1930s with rats, dogs, monkeys, and humans which were all conducted with what has become the standard US for EBCC particularly in humans – a puff of air to the eye (50). The first documented studies of EBCC to investigate psychiatric disorders were published in the 1950s by Spence and Taylor when EBCC was assessed in subjects with anxiety (51) and those with neurosis and psychosis (52, 53).

The first report of EBCC in patients with PTSD was a study by Ayers and colleagues using delay conditioning in veterans (54). A number of other studies followed mostly in veterans (55–58) and one in civilians (59). The consensus of these studies is that there may be changes in EBCC as a result of PTSD but the effects are quite variable and may involve personality traits (57). These studies are reviewed in more detail in the accompanying articles from the Servatius laboratory.

### EBCC in animals

As noted above, the history of EBCC in animals began with studies using dogs in 1935, monkeys in 1936 (50), and rats in 1938 (60). Perhaps because of the strong focus on human eyelid conditioning in the intervening years (48), little if any attention was paid to EBCC in animals until the 1960s. A return to EBCC in animals may also have reflected the neurobiological limitations inherent in and the growing theoretical and methodological controversies surrounding human EBCC (47, 61, 62). To address these methodological issues as well as provide the behavioral basis for studying learning’s neural substrates, Gormezano and colleagues developed classical conditioning of a series of related skeletal responses in the rabbit centered on the eyelid and nictitating membrane (63–66). These preparations were followed by the development of jaw movement conditioning, classical conditioning of an appetitive response (67), and heart rate conditioning, classical conditioning of an autonomic response (68, 69). In order to overcome the very limited ability to use invasive techniques in humans and pursue the growing interest in the neural substrates of learning, Thompson and colleagues began to use neural recording and lesion techniques to delineate the pathways and substrates of EBCC in the rabbit (70–72).

## Reflex Modification

**Figure 1:**
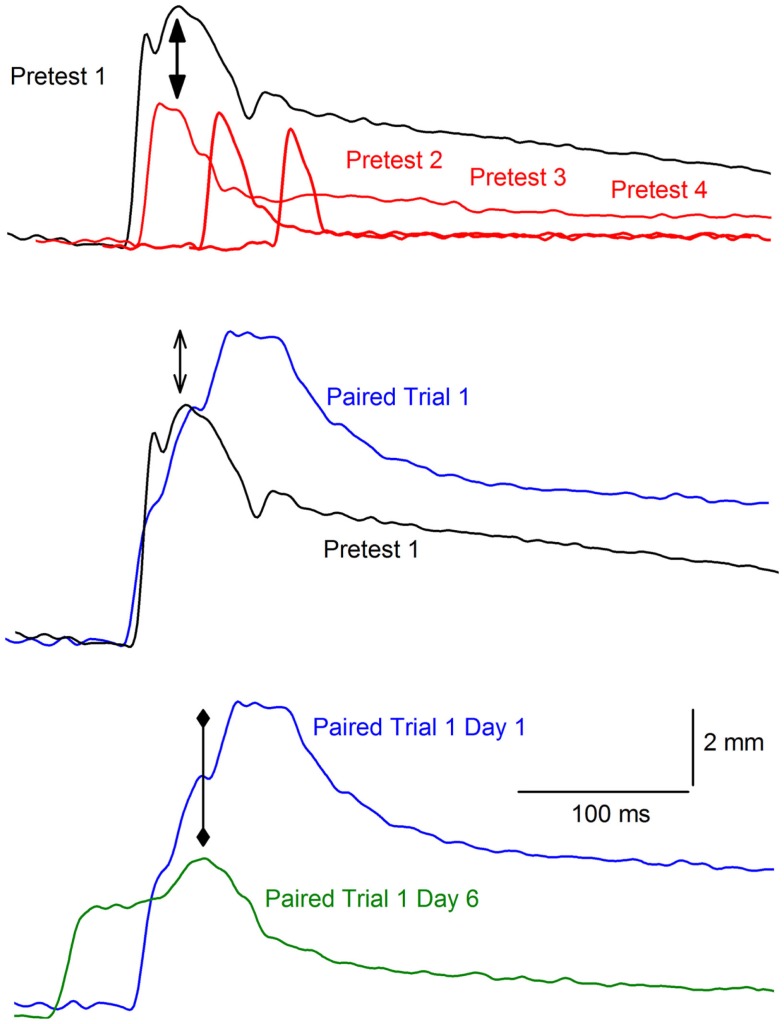
**Example of habituation and reflex modification**. The top panel of the figure shows representative nictitating membrane responses (eyeblink) to a 2.0-mA, 100 ms periorbital electrical stimulus for an individual rabbit during the first (black, Pretest 1), second (red, Pretest 2), third (Pretest 3), and fourth (Pretest 4) block of pretesting to periorbital electrical stimuli of different intensities (0.1, 0.25, 0.5, 1.0, and 2.0 mA) and durations (10, 25, 50, 100 ms). The onset of the responses are staggered from left to right to help illustrate the decrease in response amplitude (solid arrows) known as habituation as a function of repeated stimulus presentations across the four blocks. The middle panel shows the response on Pretest 1 (black) compared to the response to the same 2.0-mA, 100 ms periorbital electrical stimulus on the first paired trial (blue, Paired Trial 1) of the tone conditioned stimulus and the periorbital electrical unconditioned stimulus. The open arrows indicate the increase in the amplitude of the response known as reflex facilitation on the paired trial. The bottom panel depicts the response on the first paired trial of the tone conditioned response and the periorbital electrical unconditioned stimulus on the first day (blue, Paired Trial 1 Day 1) compared to the first paired trial on the sixth and last day (green, Paired Trial 1 Day 6). The diamond arrowheads indicate the decrease in the amplitude of the response on the later paired trial when a conditioned response is present (earlier response onset). This decrease in amplitude is known as conditioned diminution.

Although the focus of nearly all classical conditioning experiments has been on the development of a CR (e.g., eyeblink) to the conditioned stimulus (CS, e.g., tone), some attention has also been paid to the unconditioned response (UR, e.g., eyeblink) to the US. For example, there is ample evidence that URs may be modified as a result of non-associative processes. Illustrated in the top panel of Figure 1 is an example of a non-associative change in the eyeblink where repeated elicitation of the eyeblink indexed by measuring the nictitating membrane response (NMR) can lead to a reduction in the amplitude of the response known as habituation (73–81). In this example, a rabbit’s response to a strong periorbital electrical stimulus (2 mA, 100 ms) decreases across four 20-trial blocks of electrical stimulation presented at different intensities (0.1, 0.25, 0.5, 1.0, and 2.0 mA) and durations (10, 25, 50, 100 ms). URs may also be enhanced or undergo sensitization; that is, a response to a weak stimulus will become larger if it is elicited after a series of stronger stimulations (82). Although non-associative, sensitization can also occur during pairings of the CS and US and can be estimated on the basis of unpaired presentations of these two stimuli (83). A CS may facilitate the rabbit NMR the first time the tone and air puff (or periorbital electrical stimulation) are presented together (that is, before any association could have formed between the two stimuli). Depicted in the middle panel of Figure 1 is an example of an eyeblink that increased in size in the presence of a tone CS – a phenomenon known as reflex modification, in this case reflex facilitation (84–96).

Unconditioned responses may also be modified as a result of associative processes and there is substantial evidence that a UR can be modified as a function of CS–US pairings. For example, the presence of a CS may decrease the size of the UR after repeated pairings have resulted in the formation of an association. This is a phenomenon known as conditioned diminution (85, 89). The bottom panel of Figure 1 shows an example of conditioned diminution where there is a decrease in the amplitude of the eyeblink UR from the first paired trial where there is no CR to a later paired trial where there is a CR (indicated by the earlier onset latency compared to the first trial on which only a UR is present).

In all of these aforementioned studies, the focus has been on changes in the UR that are attributable to the CS. Consequently, dependent variable measures, such as amplitude of the response, have been assessed in the presence of the CS as in the case of the bottom panel of Figure 1. Our original studies were influenced by the hypothesis that classical conditioning alters not only CS processing but also alters US processing. This hypothesis is consistent with a local interaction model of learning and memory in which CS and US inputs interact at a number of local dendritic sites distributed across a neuronal array (97, 98). It is from this background that we first observed the changes in the UR that has come to be termed CRM (37). By way of contrast to earlier studies where the UR was assessed in the presence of the CS, the experiments reviewed here focus on the effects of conditioning on responding to the US in the absence of the CS and, hence, examined conditioning-specific effects that are intrinsic to US processing and UR production.

## Conditioning-Specific Reflex Modification

### The basic phenomenon

**Figure 2:**
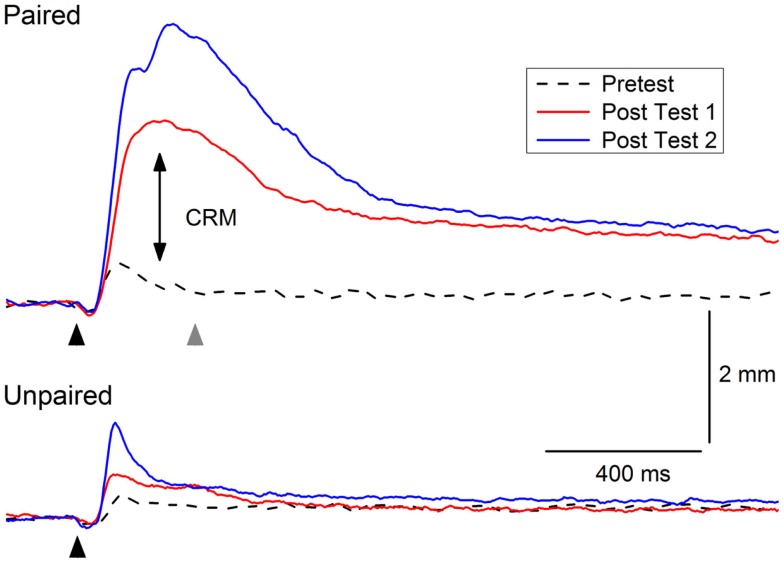
**Example of conditioning-specific reflex modification (CRM)**. Representative nictitating membrane responses (eyeblink) to 0.5-mA periorbital electrical stimulation (black arrowhead) averaged over four durations (10, 25, 50, 100 ms) in an individual rabbit before (dashed line, Pretest), 1 day after (red line, Post Test 1), and 1 month after (blue line, Post Test 2) 6 days of conditioned stimulus–unconditioned stimulus pairings (eyeblink classical conditioning, Paired). The responses show clear increases in amplitude, area, and peak latency (double arrow, CRM) compared to the responses of a control rabbit to 0.5-mA periorbital electrical stimulation (black arrowhead) averaged over four durations (10, 25, 50, 100 ms) before (dashed lined, Pretest), 1 day after (red line Post Test 1), and 1 month after (blue line, Post Test 2) 6 days of explicitly unpaired presentations of the conditioned stimulus and unconditioned stimulus (Unpaired). The gray arrowhead indicates where a 2.0-mA shock would have occurred during conditioned stimulus–unconditioned stimulus pairings. Although there is a slight increase in the amplitude of the response in the rabbit in the Unpaired group 1 month after explicitly unpaired presentations of the conditioned stimulus and unconditioned stimulus, it is not as large as the response seen in the rabbit from the Paired group nor is there a shift to the right in the peak latency.

Figure 2 shows an example of CRM in which representative NMRs to a 0.5-mA periorbital electrical stimulus are shown in a rabbit before (Pretest), 1 day after (Post Test 1), and 1 month (Post Test 2) after 6 days of EBCC (Paired). The responses show clear increases in amplitude, area, and peak latency compared to the responses in a control rabbit after 6 days of explicitly unpaired presentations of the tone CS and periorbital electrical stimulation US (Unpaired). Thus, CRM occurs following EBCC and persists for a month but does not occur following explicitly unpaired stimulus presentations – the optimal control condition for assessing non-associative contributors to responding (83). CRM is detected by comparing responses to a range of US intensities presented by themselves before and after classical conditioning and has been observed by others following EBCC in rabbits (44, 45) and rats (46). CRM is not idiosyncratic to EBCC because we have also found CRM of heart rate as a result of heart rate classical conditioning (42, 99, 100). Thus, the effect appears to exist in at least two species and in both the autonomic and the skeletal response systems. Given the subject of the present focus topic, this review will be limited to changes in the rabbit unconditioned NMR that occur as the result of EBCC because CRM of HR is obtained at conditioning parameters (i.e., long interstimulus intervals) that do not normally support EBCC. The NMR serves as a convenient index of the eyeblink as it is a component of the defensive response system consisting of closure of the upper eyelid, retraction of the eyeball, and sweep of the nictitating membrane which are very highly correlated (63, 65, 101).

### Behavioral laws

Rabbit EBCC has yielded a large number of behavioral “laws” that have been enumerated and detailed elsewhere (63, 66, 69, 102, 103). Chief among these “laws” is the relationship between the strength and rate of EBCC and a number of parameters including CS and US intensity and duration, interstimulus interval, and number of stimulus pairings (66). In a series of experiments reviewed previously (40, 42), we have found that CRM is also a function of a number of parameters including the nature (air puff and periorbital electrical stimulation) and intensity of the US (39, 104), the interstimulus interval (105), and the number of pairings (37, 38).

### Stimulus generalization

Another important phenomenon in rabbit EBCC that has been observed in other species and behavioral paradigms is generalization – responding to stimuli similar to the stimulus used during EBCC (106–108). CRM by its very nature is generalization along the intensity dimension of the US for both electrical stimulation and air puff (39). Due to a ceiling effect for the highest intensities of periorbital electrical stimulation, the strongest levels of CRM are detected below the training intensity (37–39). This is not the case for the weaker stimulation afforded by air puff where CRM occurs at high as well as moderate stimulus intensities (39). We have found that CRM can generalize from periorbital electrical stimulation to air puff but does not generalize from air puff to periorbital shock which seems to reflect the need for an intense US to support CRM (39) making it relevant for modeling PTSD.

### Context

Previous experiments suggest that CRM obeys behavioral laws similar to those of classical conditioning and, like classical conditioning, CRM is sensitive to a shift in context (41). In a series of experiments the auditory, olfactory, tactile, and visual properties of the context in which rabbits were given EBCC and CRM testing were manipulated to determine the effects of context on the level of CRM. An initial experiment demonstrated that when CRM was tested in a novel context, CRM levels were as strong as when testing occurred in the familiar, EBCC training context. To factor out differences in the amount of exposure to the different contexts that may have explained the results of the first experiment, exposure to all contexts was equated in a second experiment. The results showed that there was less CRM when testing took place in a context that was equally familiar but different from the EBCC training context. A context-dependent reduction in responding during EBCC has been demonstrated in rabbits that showed a drop in conditioned responding of 50% when given pairings in a different context where the visual, tactile, and olfactory characteristics had been altered from the original training context (109). The reduction in responding as a result of a context shift during rabbit EBCC has been reported in other learning paradigms including fear conditioning (110, 111), taste aversion learning (112), and conditioned suppression (113). Consistent with this context shift effect, our context experiments show that if exposure to the contexts is equated (111), CRM can be significantly reduced, but not eliminated, by a shift in the context from training to testing.

### Resilience and susceptibility

**Figure 3:**
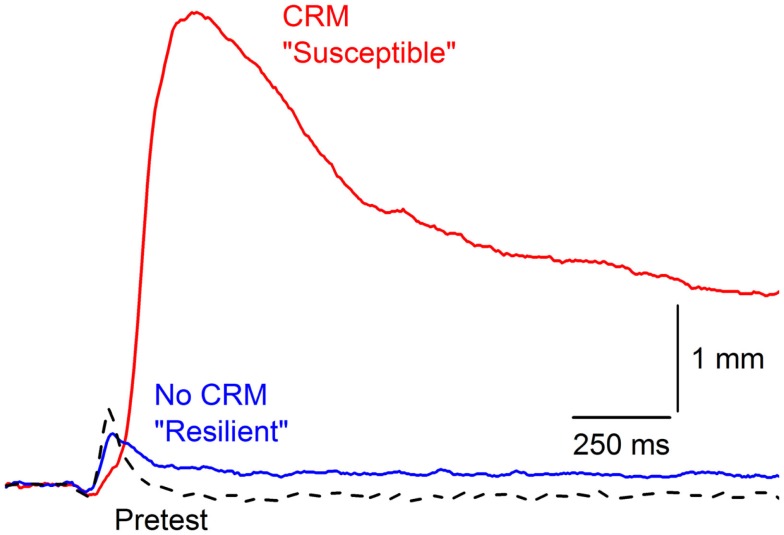
**Susceptibility and resilience of conditioning-specific reflex modification**. Representative nictitating membrane responses (eyeblink) to 0.5-mA periorbital electrical stimulation averaged over four durations (10, 25, 50, 100 ms) in two individual rabbits that show very different responses to the same 0.5-mA US on Post Test after having been given 6 days of conditioned stimulus–unconditioned stimulus pairings (eyeblink classical conditioning) at the same time to essentially the same high levels (100 vs. 98.5% conditioned responses). The rabbit with the larger, later response (red line) is considered “Susceptible” with a 2000% increase in response amplitude whereas the rabbit with a response that did not differ from Pretest is designated “Resilient” (dashed line). Figure adapted from Smith-Bell et al. (43), Copyright 2012 by the American Psychological Association.

Examination of individual subject data across CRM studies revealed CRM is not an all-or-none phenomenon with considerable between-subject variability in the presence and degree of CRM. Although some CRM occurs in over 50% of rabbits, high levels of CRM (one standard deviation above mean percent change) only occur in 15–25% of rabbits even though all reach conditioning levels in excess of 85% CRs. Figure 3 shows an example of the extremes in responding by two different rabbits to the same 0.5-mA periorbital electrical stimulus. Despite high, almost identical levels of EBCC (100 vs. 98.5% CRs), these two subjects show profound differences in their responses to the periorbital electrical stimulus on Post Test. The first subject shows particularly strong CRM and would be considered “susceptible” whereas the second subject shows no CRM at all and would be considered “resilient.” In 135 subjects trained with our standard EBCC paradigm consisting of 80 daily presentations of a 400-ms, 82-dB, 1,000 Hz tone CS that coterminates with a 100-ms, 2.0-mA, 60-Hz periorbital electrical stimulus, we found the strongest predictor of CRM (indexed by an increase in response magnitude and area) was short CR onset latency (43). We also found that during periorbital electrical stimulation on Pretest, the strongest predictor of subsequent CRM was response onset and peak latency – the faster the rabbit’s response, the more likely it was to develop CRM. Therefore, the speed with which a rabbit responds to the CS during training and to the periorbital electrical stimulus during pretest are good predictors of CRM and are indices of susceptibility. This would correspond to differences in reaction time in PTSD – something that is not often observed (114–116) but has been reported (117).

### Incubation

**Figure 4:**
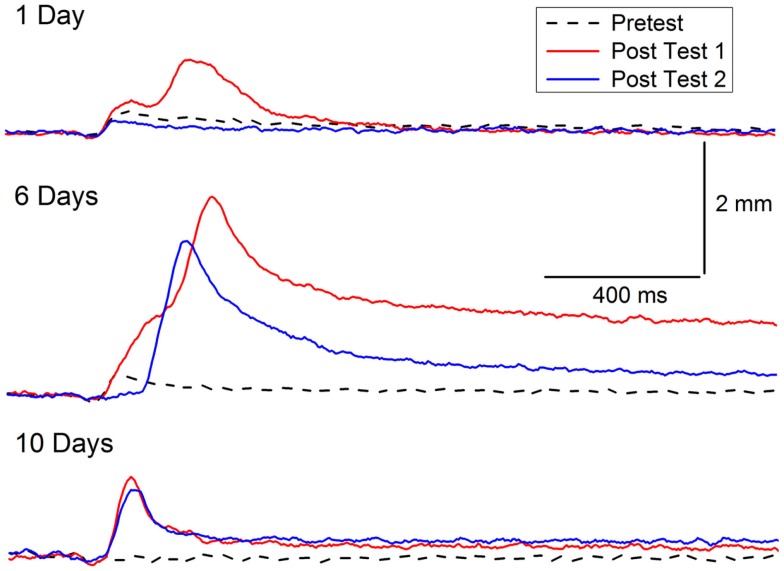
**Incubation of conditioning-specific reflex modification**. Representative nictitating membrane responses (eyeblink) to a 0.25-mA periorbital electrical stimulus averaged over four durations (10, 25, 50, 100 ms) in individual rabbits before (dashed line) and after 1, 6, or 10 days of incubation in the home cage (red line) following a single session of conditioned stimulus–unconditioned stimulus pairings (eyeblink classical conditioning) which supported a level of only 45% conditioned responses. The blue lines depict nictitating membrane responses to 0.25-mA periorbital electrical stimulation 7 days (of incubation) after Post Test 1. Although there is some suggestion of conditioning-specific reflex modification after 1 and 10 days of incubation, there was very clear and strong conditioning-specific reflex modification that occurred after 6 days of incubation and persisted a week later. Figure adapted from Schreurs et al. (126), used with permission from Elsevier.

The symptoms of PTSD do not always occur immediately after trauma and can become more pronounced over time. A delay in the onset of symptoms by as much as 6 months has been incorporated into previous diagnostic criteria of PTSD (118, 119), but there is now debate about whether delayed-onset PTSD actually exists in either veterans or civilians with evidence for both points of view (118–124). In our animal model of PTSD symptoms, rabbits do not show a delay in onset of CRM, but there is a window during which incubation exacerbates CRM. The results are consistent with clinical data in which exacerbation or reactivation of prior symptoms accounts for 38.3% of military cases of PTSD and 15.3% of civilian cases (120, 125). In one set of experiments, we have observed the exacerbation of symptoms as a function of a period of incubation (126). CRM typically requires at least 3 days of EBCC when levels of conditioning reach or exceed 85% CRs (37, 39). We carried out an experiment (Figure 4) in which rabbits were given EBCC for just 1 day resulting in mean conditioning levels of only 45% CRs, and saw little evidence of CRM when tested the next day. However, if left in their home cages for 6 days, there was a significant amount of CRM which persisted for a week after testing (126). The incubation effect was not strong following 10 days in the home cage and did not persist. These data suggest there may be no delay in CRM onset but there is a window for incubation to exacerbate CRM.

### Response generalization

**Figure 5:**
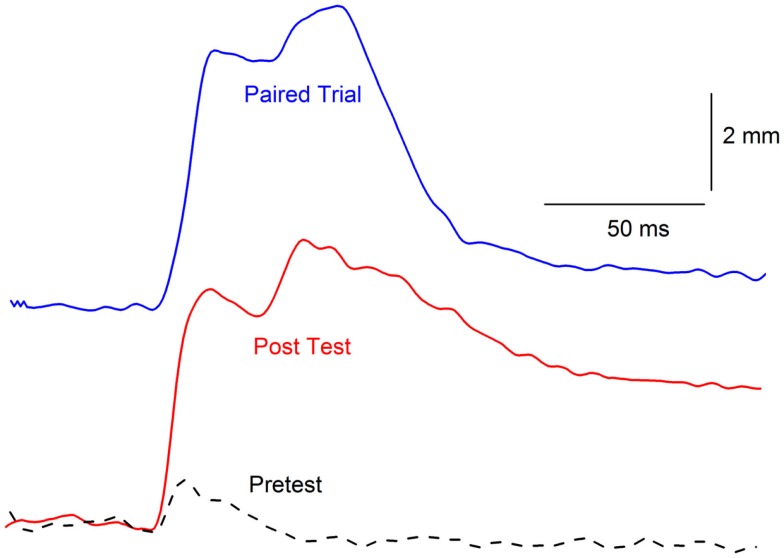
**Topographical similarity between a conditioned response and conditioning-specific reflex modification**. Representative nictitating membrane responses (eyeblinks) in the same rabbit to a tone paired with shock during the third day of conditioned stimulus–unconditioned stimulus pairings (eyeblink classical conditioning, blue line, Paired Trial) and 0.5-mA periorbital electrical stimulation presented by itself before (dashed line, Pretest), and after (red line, Post Test) 6 days of conditioned stimulus–unconditioned stimulus pairings. The response after eyeblink classical conditioning shows a strong similarity in response amplitude, peak latency, and overall topography compared to the response before eyeblink classical conditioning. The responses are shifted in time so that their onsets coincide even though the response on the paired trial is to the conditioned stimulus that overlaps with the periorbital electrical stimulus and the responses on the Pretest and Post Test trial are to 0.5-mA periorbital electrical stimulation by itself.

**Figure 6:**
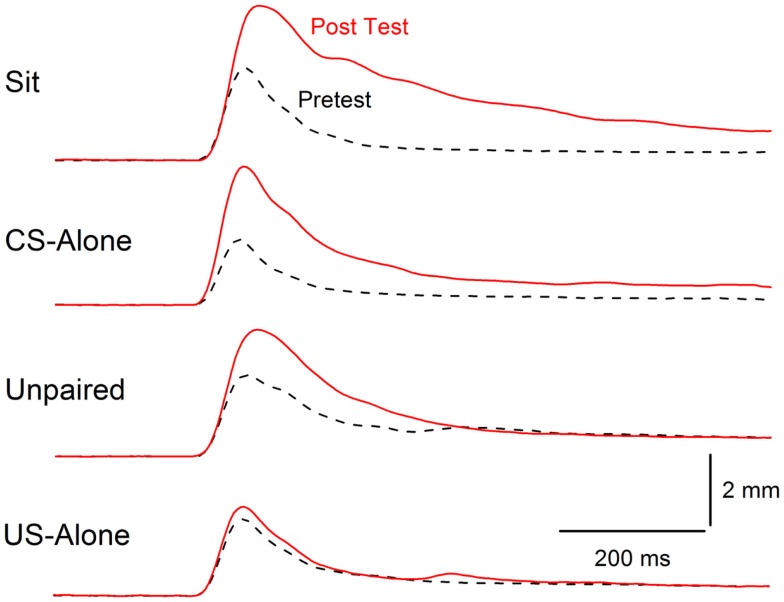
**Extinction of conditioning-specific reflex modification**. Averaged nictitating membrane responses (eyeblink) to a periorbital electrical stimulus of 1.0 mA averaged over four durations (10, 25, 50, 100 ms) for four groups of rabbits before 6 days of conditioned stimulus–unconditioned stimulus pairings (eyeblink classical conditioning) (dashed lines, Pretest) and 6 days after (red line, Post Test) either exposure to the training chamber with no further stimulus presentations (Sit), presentations of the conditioned stimulus alone (CS-alone), explicitly unpaired presentations of the conditioned stimulus and unconditioned stimulus (Unpaired), or presentations of the unconditioned stimulus alone (US-alone). The level of conditioning-specific reflex modification (CRM) was highest in the Sit group, followed by the CS-alone group, and the Unpaired group with virtually no CRM in the US-alone group. Although there was residual CRM in the Unpaired group, rabbits in this group showed no responding to the conditioned stimulus unlike rabbits in the Paired and US-alone groups suggesting that only unpaired presentations of the conditioned stimulus and unconditioned stimulus were able to significantly reduce CRM as well as eliminate conditioned responses to the conditioned stimulus. Figure adapted from Schreurs et al. (38), in the public domain.

One of the most interesting aspects of our initial CRM experiments was the observation that, in individual subjects, responses to weak periorbital electrical stimulus intensities appeared to have a significantly different topography after EBCC than they do before EBCC and that the topography was reminiscent of the CR (37, 40). This observation was even more clearly articulated by Gruart and Yeo (44) when they first reported changes in the rabbit eyelid UR following EBCC. The marked alteration in response topography is somewhat lost in the averaging that takes place when presenting group data especially when, as noted above, not all rabbits show CRM. Figure 5 shows the strong similarity between a CR that occurs during EBCC and a UR to periorbital stimulation by itself assessed after EBCC compared to an UR assessed before EBCC. These early observations lead to the hypothesis that CRM is a CR that generalized from the CS–US pairings to the US itself (40, 44). A series of experiments were conducted to test this hypothesis by altering the topography of the CR by presenting two shocks during CS pairings or by presenting CS–US pairings with two different interstimulus intervals (38). The results provided evidence both for and against the hypothesis so a final experiment was designed to eliminate CRs by presenting the CS by itself during extinction (38). If the exaggerated responses to the US after EBCC (CRM) were generalized CRs, it was reasoned that eliminating the CRs should eliminate CRM. The results of this experiment were more conclusive. Despite reducing CRs to essentially baseline levels of less than 10% by presenting the CS by itself, Figure 6 shows CRM remained virtually intact. A number of control groups actually proved to be even more instructive. First, presentations of the US by itself completely eliminated CRM as shown in Figure 6 but left CRs relatively intact. Thus, the extinction of CRs left strong levels of CRM and the extinction of CRM left strong levels of conditioned responding. Second, combining presentations of the CS and the US in an explicitly unpaired manner resulted in elimination of CRs and a reduction in the level of CRM (Figure 6). It was these experiments that led to a further exploration of treatments that eliminate both CRs and CRM as a possible treatment strategy for PTSD.

### Extinction of CRM

There is a significant body of evidence from both clinical and basic research that repeated presentation of feared stimuli does not prevent fear from returning – a phenomenon referred to as “relapse” (127, 128). Nevertheless, fear extinction is a cornerstone of many approaches to the treatment of PTSD (3, 28, 129–137). However, the renewal of fear or relapse may be “thwarted” by unpaired presentations of both the feared stimulus and the event producing the fear (38, 138–140). Experiments drawn from a large number of different conditioning paradigms including human and rabbit EBCC (36, 39, 141–144), as well as conditioned bar-press suppression in rats (138, 139), and human discriminative fear conditioning (140) show unpaired presentations of the CS and US produce extinction of a CR. In the human discriminative fear study, Vervleit and coworkers found that compared to normal extinction, only unpaired extinction prevented renewal of fear responses in people trained to discriminate one of two pictures paired with shock (140).

In rabbit experiments designed to extinguish EBCC, comparable extinction of responding to the CS occurs following CS-alone or unpaired CS and US presentations (38). However, as noted above and shown in Figure 6, unpaired presentations were able to extinguish CRM better than CS-alone presentations (38). The ability of unpaired presentations to diminish both CRs and exaggerated URs (i.e., CRM) suggests it may be relevant for treating both the conditioned fear and hyperarousal symptoms of PTSD (41, 42, 104). However, no matter how effective unpaired extinction might be in extinguishing fear and hyperarousal in animal models, it would be ethically unacceptable for treating PTSD because the US intensity used in unpaired extinction has always been the same as that used to induce classical conditioning (36, 39, 138–144). The repeated presentation of a traumatic event responsible for PTSD in order to treat it is untenable.

## Unpaired Extinction That is Clinically Relevant

**Figure 7:**
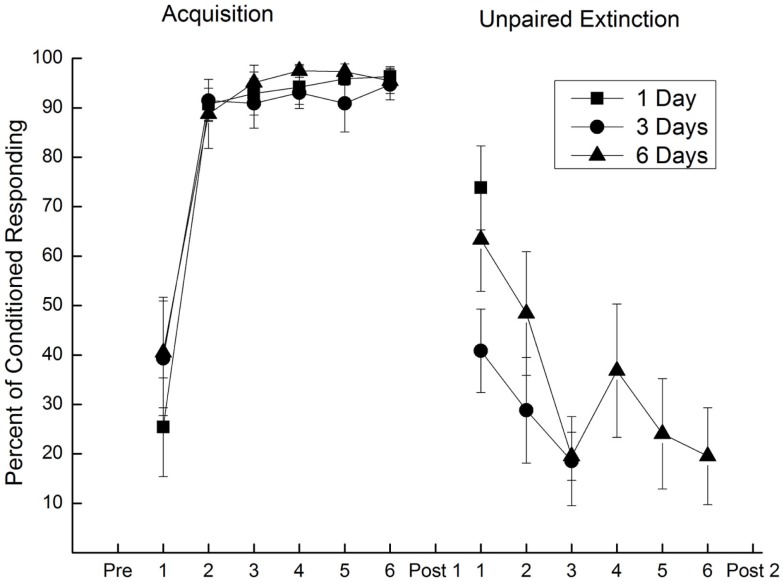
**Extinction of conditioned responding with unpaired presentations of a conditioned stimulus and weak unconditioned stimulus**. Mean (±SEM) conditioned responses to the conditioned stimulus during 6 days of pairings of a conditioned stimulus and a 2.0-mA unconditioned stimulus (Acquisition) and during one (square), three (circle), or six (triangle) subsequent days of unpaired conditioned stimulus–unconditioned stimulus presentations (Unpaired Extinction) with a 0.25-mA unconditioned stimulus. Conditioned responding increased to asymptotic levels of 95% during Acquisition and fell to less than 20% following 3 days of unpaired presentations (Unpaired Extinction). Figure adapted from Schreurs et al. (36), used with permission from Elsevier.

**Figure 8:**
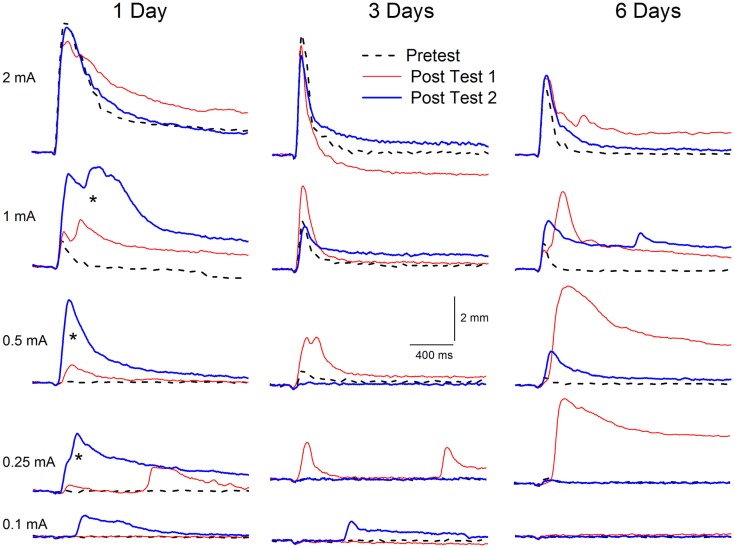
**Extinction of conditioning-specific reflex modification with a weak unconditioned stimulus**. Representative nictitating membrane responses (eyeblink) to a range of periorbital electrical stimulus intensities (0.1, 0.25, 0.5, 1.0, and 2.0 mA) averaged over four durations (10, 25, 50, 100 ms) in individual rabbits before (dashed line) and 6 days after (red line) conditioned stimulus–unconditioned stimulus pairings (eyeblink classical conditioning), and again after (blue line) 1, 3, or 6 days of explicitly unpaired stimulus presentations of the conditioned stimulus and a weak unconditioned stimulus (0.25 mA) that was 87.5% weaker than the periorbital electrical stimulation previously used to establish eyeblink classical conditioning (2.0 mA). Conditioning-specific reflex modification (CRM) was established as a result of the 6 days of eyeblink classical conditioning [comparison of Pretest (dashed lines) to Post Test 1 (red lines) for intensities below the training intensity of 2.0 mA]. Following as few as three subsequent days of explicitly unpaired stimulus presentations of the conditioned stimulus and weak unconditioned stimulus, the levels of CRM (Post Test 2, blue lines) were all lower than after conditioning (Post Test 1, red lines) and in many cases returned toward baseline levels (Pretest, dashed lines). Importantly, the level of CRM increased significantly (asterisks) after just 1 day of explicitly unpaired stimulus presentations of the conditioned stimulus and weak unconditioned stimulus. Figure adapted from Schreurs et al. (36), used with permission from Elsevier.

To address concerns about using a traumatic stimulus during unpaired extinction and make an unpaired extinction procedure more clinically relevant, rabbit EBCC experiments were conducted in which unpaired extinction sessions employed periorbital electrical stimulation of reduced intensity that was presented for different numbers of days (36). Specifically, rabbits received US testing (Pretest), EBCC, another session of US testing to determine the size of CRM (Post Test 1), and then 1, 3, or 6 days of unpaired CS and US presentations with a weak (0.25 mA), moderate (1.0 mA), or strong (2.0 mA) US followed by a final session of US testing to determine the effect of unpaired presentations on CRM (Post Test 2). The results revealed extinction of both CRs and CRM was a function of the US intensity used during unpaired stimulus presentations and the number of days of those unpaired stimulus presentations (36). The levels of CRs declined from 95% to less than 20% within 3 days of unpaired stimulus presentations. Figure 7 shows CRs during acquisition and 1, 3, or 6 days of unpaired extinction in which the US intensity was eight times weaker (0.25 mA) than the intensity used during pairings (2.0 mA). Figure 8 depicts sample responses from different rabbits before and after EBCC (Pretest and Post Test 1, respectively) and again after unpaired stimulus presentations (Post Test 2) with a 0.25-mA US that were delivered for either one, three, or six daily sessions (days). The sample responses in the middle and right illustrate that after as few as three sessions of unpaired presentations with a weak US, any CRM seen after EBCC (red lines) was largely eliminated (blue lines). In contrast, the sample responses on the left show clearly that CRM was actually enhanced after a single session of unpaired presentations with a weak US. Taken together, these data suggest that both CRs and CRM seemed to be diminished, if not eliminated, most effectively with at least 3 days of mild US presentations but one session of stimulus presentations actually appears to exacerbate responding. Of note, and of particular clinical relevance, was the finding that extinction of CRs and CRM occurred even though the weak US produced relatively low levels of responding (rabbits blinked to the weak US on less than 25% of occasions). Analysis of rabbit heart rate during these sessions indicated that this weak US did not produce any change in heart rate, suggesting it was not unduly stressful (36). One important implication of these data is that treatment must not be brief because brief treatment using unpaired stimulus presentations may not just be ineffectual; it may actually heighten the symptoms of PTSD.

## Virtual Reality

If weakened versions of the initiating trauma are to be used as part of PTSD therapy, there would be very few such events that could or even should be repeated or recreated. The advent of credible virtual reality (VR) environments that have been developed to treat PTSD provide a feasible way around this stricture (145–151). Given the unpaired extinction data reviewed above, one could imagine a treatment situation in which a PTSD patient could be asked to describe a specific trigger or set of triggers for unwanted memories (150) and present the trigger(s) in an unpaired manner with a weakened version of an aversive event. A weakened but still stressful version of an explosion might be strongly shaking a driver’s seat in a virtual Humvee which is part of a VR scenario in which the sights and sounds of combat are also presented (149–151). The VR environment could be programed to present these events in a separate, unpaired manner and the prediction would be that, with a number of repetitions over more than one session, PTSD symptoms would abate. For example, the sights, sounds of a previously traumatic context could be presented, and then the goggles and headphones would go blank and silent for a period of no stimulation which would then be followed by the driver’s seat being strongly shaken. The sequence of these series of events would be randomized so that they would never occur together to reflect the explicitly unpaired procedure (83). Importantly, given that CRM has been shown to generalize from stressful periorbital electrical stimulation to what would be considered less stressful air puffs, the weakened versions of stressful events used in an unpaired extinction procedure may not need to involve the traumatic event. Psychophysiological indices including heart rate, skin conductance, respiration, and cortisol levels could be used to assess stress levels and titrate the intensity of the stimulation.

## Methodological Addendum

### Stimulus delivery and response measurement

The experiments described in this review require precise control and calibration of stimulus parameters particularly intensity and timing of the US. This is relatively straightforward for periorbital electrical stimulation through the use of programmable shock delivery equipment and the use of digital computer control. On the other hand, the delivery of air puff requires more elaborate equipment and techniques including a digitally controlled, programmable pressure regulator and an accurate digital manometer to ensure that the intensity of the air puff reflects the air striking the cornea and not the pressure at the source. Response detection is also of importance especially if response characteristics such as latency, amplitude, and area are to be determined in addition to simply registering if a response occurs or not. As a result, transduction and recording of the eyeblink response becomes important. Researchers may wish to consider the advantages and disadvantages of remotely sensing versus directly measuring the closure of the eyelid using mechanical coupling. For example, infrared reflectance measures may not be capable of completely quantifying the peak latency of a response whereas mechanical couple may produce drag that subtly alters the latency and amplitude of a response (152). EMG recording of the *orbicularis oculi* muscle may have advantages but the electrical noise induced by periorbital electrical stimulation as well as time constants of integration affecting onset latency and difficulty in determining units of response amplitude present limitations in quantifying the UR.

### Data analysis

Even if the UR is transduced accurately, questions remain about the analysis of data, particularly when responses are at the limits of detectability as the result of very weak stimulation. By convention and due, in part, to the limits of analog instrumentation, an NMR or eyeblink response has been defined as movement of at least 0.5 mm (61, 66, 153, 154). How then is a change in response amplitude and latency from pretest to post test determined if there is no response on pretest but a significant response on post test as often occurs after EBCC? The main issue has always been what to do about the lack of a response on pretest or post test. We have addressed this in several ways including analyzing individual subject data only for US parameters at which responses occurred (37–39, 104), averaging topographies across subjects and analyzing for changes in skew and kurtosis (41, 155), and calculating percent change where a response on a test was considered to be a 100% change if there was no response on the other test (43). Most recently, two additional measures, magnitude of the response and magnitude of the response area, have been calculated to overcome the limitations of empty data cells on pretest or post test resulting from subthreshold URs, particularly at lower US intensities and durations (36, 43, 126). Magnitude of the response and magnitude of the response area have included the amplitudes and areas of all nictitating membrane movements above baseline and provide the most procedurally neutral estimates of responding (154).

### Conditioned response definition

Another issue in data analysis turns upon the practice of categorizing responses as CRs if they are “adaptively timed,” a term based on the onset latency of responses (this is probably wrong anyway because one should be looking at the latency of the peak to coincide with US delivery but that would require CS-alone test trials that are un-confounded with the UR to the US which many experiments do not include). The concept of adaptively timed responses is based on the notion that CRs lessen or even avoid the aversiveness of the US when the maximum closure of the eyelid coincides with the occurrence of the US. This adaptive response may therefore be argued as being reinforcing, adding an instrumental component to CRs also known as the “law of effect” (156–158). Coleman has reviewed the literature on the “law of effect” and conducted an experiment showing quite clearly that, at least in rabbit EBCC, the imposition of a contingency between the occurrence of a CR and a reduction in the intensity of a shock US results in less rather than more responding – a finding that completely contradicts a “law of effect” prediction (156). In other experiments, including tail flexion in the rat (159), appetitive jaw movement conditioning in rabbits (160) and human EBCC (157), the lack of significant effect and even inferior conditioning of subjects explicitly designed to benefit from the “law of effect” is clear (157, 159, 160). In contrast, early experiments by Schlosberg were interpreted as “successful” only if CRs modified the US (60, 161). In fact, Schlosberg used the term “adaptive” in describing responses that had an effect on the US and “non-adaptive” for those that did not (p. 383). The pervasiveness of this assumption about the “role” of the occurrence and timing of CRs and its periodic reintroduction (162) may account for more modern EBCC experiments in which responses are only considered to be CRs if they occur within an interval that is characterized as “adaptive.”

The use of onset latencies to detect adaptively timed CRs and hence, “true CRs” can be traced to another period in the history of EBCC where latencies were used to identify and eliminate the data of “voluntary responders” (62, 163, 164). Voluntary responders were subjects who were “rejected” from experiments based on the occurrence of short-latency eyeblinks that occurred between 200 and 300 ms after CS onset and were judged to have the same appearance as subjects who were instructed to blink or by subjects who reported they were blinking “voluntarily” to avoid the air puff (165). This practice has been explicitly adopted by a number of laboratories especially during trace conditioning where there was a long interval between the offset of the CS and the onset of the US because it “corrected for both voluntary and random blinks that could occur as a result of the longer trace intervals” (166, 167).

In our view, an empirical approach to determining onset latency needs to be neutral with respect to characterizing responses. We endorse the complete characterization of all responses using a range of dependent variables including onset and peak latency and presenting all response onsets on a latency histogram without any preconceptions of how responses should look or be distributed. Publication of such histograms together with any interpretation of what are considered responses whether they be “adaptive” or not would allow readers to interpret the data for themselves.

## Summary and Conclusion

There is a crucial need to know how responding to stressful events changes as a function of trauma for those who suffer from PTSD. A number of treatment strategies for PTSD are capable of treating only some of the symptoms because the extinction of fear does not deal with the various forms of hyper-vigilance and hyperarousal experienced by people with PTSD, especially in combat veterans. Based on our work on conditioning of the rabbit’s NMR, we have developed a preclinical EBCC model of PTSD that addresses both CRs to trauma-associated cues as well as hyperarousal (CRM). Animal models of EBCC are particularly useful here because EBCC is one of the few behavioral paradigms in which there is a one-to-one correspondence between animals and humans. We have demonstrated that CRM follows many of the same behavioral rules as EBCC, can generalize across stimulus modalities, shows sensitivity to context manipulations, and can be exacerbated after an incubation period. Importantly, CRM does not develop in all animals just as PTSD does not develop in all those exposed to trauma, with some individuals demonstrating susceptibility while others show resilience. We have shown that CRs and CRM can be simultaneously extinguished by unpaired stimulus presentations, even when US intensity is reduced to the point where it is barely capable of eliciting a response. This is important because presenting strong unconditioned stimuli as a therapeutic approach would be untenable. These unpaired procedures with attenuated stimuli have direct implications for the treatment of PTSD and could be implemented in a VR environment.

## Author Contributions

BS and LB conceived and wrote the manuscript.

## Conflict of Interest Statement

The authors declare that the research was conducted in the absence of any commercial or financial relationships that could be construed as a potential conflict of interest.
